# The influence of ethnic group composition on focus group discussions

**DOI:** 10.1186/1471-2288-14-107

**Published:** 2014-09-20

**Authors:** Nan Greenwood, Theresa Ellmers, Jess Holley

**Affiliations:** Faculty of Health, Social Care and Education, St George’s University of London and Kingston University, St George’s University of London, 2nd Floor Grosvenor Wing, Cranmer Terrace, London SW17 0RE UK

**Keywords:** Focus groups, Ethnic groups, Carer, Caregiver, Stroke

## Abstract

**Background:**

Focus groups are commonly used to explore participants’ experiences in health and social care research. Although it is suggested that having demographically homogenous groups may help put participants at ease, the evidence is sparse.

The aims of the paper are to: explore the impact of relative ethnic homogeneity and heterogeneity of focus group participants on the group discussions; improve understanding of homogeneity and heterogeneity in focus groups; suggest ways to operationalise concepts such as being ‘more comfortable’ with other focus group participants.

**Method:**

Digitally recorded focus groups were undertaken with family carers of stroke survivors and were later transcribed and analysed using framework analysis. Groups were designated as more or less ethnically homogenous. More homogenous groups included, for example, only White British or Asian Indian participants whilst more heterogeneous groups comprised a mixture of, for example, Asian, White British and Black Caribbean participants.

**Results:**

Forty-one carers participated in seven focus groups. Analysis revealed differences in discussions around ethnicity between the more or less ethnically homogenous groups. For example, participants in more ethnically homogenous focus groups were more likely to say ethnicity might influence perceptions of social care services. On the other hand, more heterogeneous groups emphasised similarity in carers’ experiences, irrespective of ethnicity. Participants in the more homogenous groups were also more likely to make potentially controversial comments relating to ethnic differences. Additionally they appeared to be more at ease with each other discussing the topic. For example, they spontaneously mentioned ethnic differences earlier in these groups.

In contrast, analysis of topics not specifically related to ethnicity, such as the difficult experiences of being a carer, produced no discernible patterns when comparing more and less homogenous focus groups.

**Conclusion:**

Considerations around focus group participant demographic homogeneity and heterogeneity are complex and these terms may be most usefully applied only in relative terms.

Data derived from more homogenous groups complement data from more heterogeneous groups providing different perspectives. Depending on the focus of the discussion, having characteristics in common, such as being a carer can override other differences.

## Background

### Focus groups

Focus groups have a long history in the health and social sciences [1] and are seen as providing a unique data source. Definitions of focus groups tend to emphasise their value in exploring participants’ knowledge and experiences and in helping to understand not only what people think, but also why they think the way they do, about a specific issue [2]. Focus group ‘participants relate their experiences and reactions among presumed peers with whom they are likely to share some common frame of reference’ ([1]:294), encouraging them to explore issues of importance to them in their own words [2]. They are particularly useful for gaining insight from minority ethnic groups [1, 3] because of their sensitivity to cultural variables [2, 4].

One of the main differences between focus groups and one-to-one interviews is the interaction between participants. Focus group participants can describe their experiences with others who they see as peers, often sharing a common frame of reference. This encourages them to comment on or even challenge each other’s view point [1]. Not only does this interaction allow participants to question each other but they can also build on others’ responses providing understanding of consensus and diversity within the group [4].

### Rationale for comparing more ethnically homogeneous with more heterogeneous focus groups

There are many sources of advice on the practicalities of setting up focus groups [4, 5]. Recommendations cover group composition, participant numbers and venues. Conditions for making participants comfortable in expressing their individual views are frequently highlighted and it is often suggested that participants should be in groups who are similar - homogenous rather than heterogeneous - in social backgrounds. Homogeneity in, for example, gender, age and ethnicity is often recommended. This similarity increases participant compatibility in turn making them more comfortable with each other. This allows them to speak more openly which makes conversation more free-flowing than in heterogeneous groups [5]. It is also suggested that sharing social and cultural backgrounds is particularly important when considering sensitive issues [4]. Indeed some authors even claim that ‘heterogeneous groups are generally undesirable’ ([6]:348). However, evidence to support the claims for the impact of participant demographic homogeneity on focus group data or guidance on how to operationalise concepts such as being more comfortable in a focus group setting is sparse.

The study described here was part of a large project looking at carers’ perceptions of services comparing five ethnic groups [7] and provided an ideal opportunity to explore the impact of participant homogeneity and heterogeneity in focus groups. A key area of exploration in the focus groups was ethnicity and services. This allowed us to investigate the impact of participant ethnic homogeneity and heterogeneity on discussions focussed on ethnicity. However, the group discussions were wide ranging and included conversations relating to general experiences as carers. This allowed us to investigate whether there was an impact of ethnic homogeneity and heterogeneity on discussions not focussed on ethnicity.

The available literature informed data analysis. For example, it is suggested that participants in ethnically more homogenous focus groups will feel more at ease with each other than those in more heterogeneous focus groups. This might be predicted to be shown by differences between the groups in unsolicited references to ethnic or cultural differences and in attempts to move the conversation away from the topic onto more neutral topics. In addition, the suggestion that conversations might be less free flowing when participants come from heterogeneous groups [5, 6] was also reflected in the analytic framework. Two contrasting interaction styles in focus groups have also been identified in the literature. These are ‘complementary interactions’, which draw out consensus amongst the group and ‘argumentative interactions’ where participants disagree or challenge each other [8]. It was assumed here that how comfortable participants are with each other might be reflected in their expressions of both consensus and challenge. For example, if participants are more at ease with each other, they may be more likely to challenge each other. The analysis therefore compared interactions on these dimensions in groups with more or less ethnically homogenous participants.

### Definitions and caveats

- Carer here refers to unpaid carers (often spouses or partners and adult children) who support someone living in the community.
- Stroke survivor refers to someone who has had a stroke.
- Social care services here refer to support services for either the carer or the stroke survivor and include services such as home care, day centres and support groups whether provided by statutory or voluntary sector services.
- Minority ethnic groups refers to ‘minority populations of non-European origin’ ([9]:445).

However, the diversity within ethnic groups [9, 10] and the fact that ethnicity is dynamic and multi-dimensional [11] needs to be acknowledged. Furthermore, people are not solely defined by their ethnic group membership [12]. Nevertheless despite their limitations, using these terms can help understand disadvantage, as long as the complexity of the terms is acknowledged [10].

- Homogeneity and heterogeneity in focus group composition related to ethnicity. Participants here were older carers of stroke survivors but the groups differed in ethnic group composition. The terms homogeneous and heterogeneous are used to refer to their similarity in ethnicity and not variations in, for example, gender. For example, a focus group where all participants self-identified as Asian Indian is classified as more homogenous than a focus group where participants identified themselves as being from several ethnic groups such as Black African, White British and Asian Pakistani.

### Aims

This article aims to explore the impact of relative ethnic homogeneity and heterogeneity of focus group participants on the group discussions; to improve understanding of homogeneity and heterogeneity in focus groups and to suggest ways to operationalise concepts such as being ‘more comfortable’ with other focus group participants.

### Ethical approval

Ethical approval was gained from the National Social Care Research Ethics Committee (ref: 12/IEC08/00).

## Method

To be included, participants had to self-identify as coming from one of five ethnic groups (Asian Indian, Asian Pakistani, Black African, Black Caribbean or White British), be aged over 45 years and either be currently or recently (in the last two years) caring for a stroke survivor living in the community. Participants also needed to speak English sufficiently well to participate in the discussions. This was explained to potential participants and they made the decision as to whether they felt able to participate in English. Carers had to have experiences of social care services either for themselves or the stroke survivor.

Participants were recruited from several voluntary organisations supporting, for example, carers or stroke survivors. The research team initially contacted the organisations, described the study and asked if they were able to facilitate recruitment. If they agreed, the organisations either distributed information about the study themselves or a member of the research team visited and described the study to potential participants. Participant anonymity and confidentiality were highlighted. Sampling was purposive and to include carers from five ethnic groups (Asian Indian, Asian Pakistani, Black African, Black Caribbean, White British) aged over 45 years.

Recommendations for the size of focus groups vary but typically between four and eight participants [2] are suggested. Following these recommendations, and allowing for dropouts, between five and ten participants were invited to each group.

Focus groups were facilitated by a member of the research team with experience of moderating focus groups. Another research team member took notes. A topic guide was followed ensuring that topics important to the research question were covered but also allowing additional issues to be raised. After an initial introduction, the topics explored included: experiences of social care services; examples of satisfactory and unsatisfactory services; cultural or ethnic backgrounds and experiences and satisfaction with services.

All participants were provided with participant information sheets and gave written informed consent both for audio recording of the focus groups and publication of findings and anonymised quotes. A copy of the written consent form is available for review.

Participants were not paid for attending the group but to recognise their contribution and time, they were given a retail gift voucher as a ‘thank you’ [4]. Some participants were expected to have to pay for respite during the focus groups so they were offered respite and travel expenses.

### Data analysis

Researchers using focus groups have been criticised for focussing on the content as opposed to interaction in focus groups [4, 13, 14]. However, initial scrutiny of the focus group data here revealed the difficulties in separating these two aspects of the data. For example, although the content of a joke is important, jokes can be an attempt to defuse an awkward situation thereby influencing interaction. To permit analysis of both content and interaction, focus groups were therefore transcribed verbatim (any identifying features such as names were changed) with laughter, long pauses and occasions where there was general agreement amongst participants identified in the transcripts. Noting these non-verbal aspects of the groups was intended to help understand interaction amongst participants making it possible for analysis to include a mixture of what was said, how it was said and how other participants responded.

Data were entered into NVivo 10 and analysed using framework analysis. This approach sits within thematic analysis – a broad type of analysis that identifies commonalities and differences in qualitative data [15]. Originally developed by Ritchie and Spencer (1994) [16] framework analysis is now widely used in health research [17].

Following the guidance of Ward et al. [17], one researcher (NG) led in devising the framework whilst consulting with other team members but all team members immersed themselves in the transcripts reading and re-reading them familiarising themselves with the content but also looking for indicators of how comfortable participants were in discussing ethnic differences, for example, in stilted conversation and unsolicited comments about ethnic differences.

Framework analysis permits both inductive and deductive analyses making it useful here as there were specific features of the data we wanted to explore, but it also allowed us to remain sensitive to aspects of the data not derived from the literature [18]. A mixture of deductive and inductive analysis therefore guided the development of a framework which was based both on focus group literature and on themes identified during initial data analysis.

Framework analysis permits identification of further themes developed inductively from the data. During this process three other relevant themes were identified and analysed. These included comments that were: potentially ‘controversial’ ideas relating to ethnic differences that might be perceived as discriminatory; comments emphasising the importance of good quality care for everyone irrespective of their ethnic or cultural background; descriptions of negative emotions or difficult experiences as carers.

Towards the end of the focus groups, the moderator asked a direct question about ethnicity and perceptions of social care. Responses to this question were used to focus the analysis allowing the interview transcripts to be explored to detect similarities and differences in how each group responded to the question and overall how they discussed ethnicity. Researchers’ notes were used primarily to help provide context for the focus groups and the manner of the interaction.

NG developed these themes into a framework for analysis and second researcher (TE) then used the framework on a sample of transcripts. The authors then met and discussed the framework. There was considerable consensus and the few differences were resolved by discussion and the framework was modified slightly. The framework was then used to analyse all transcripts which was undertaken independently by the authors. Table 1 describes the themes in the framework and gives examples of the evidence in transcripts which support them.

**Table 1:** **Themes in the framework**

Theme	Description of themes and evidence from the transcripts
Consensus	**Description:** Expressions of agreement in opinion with other participants.
	**Evidence:** E.g. ‘I agree’; ‘yes’; repeating what an earlier participant said; rephrasing what another participant said; completing another participant’s sentence; adding similar comments in agreement.
Challenges	**Description:** Expressions of disagreement with other participants.
	**Evidence:** E.g. ‘I disagree’; offering a counter or different examples.
Unsolicited comments about ethnic and cultural differences	**Description:** Unsolicited comments about ethnic and cultural differences.
	**Evidence:** Spontaneous comments about the impact of ethnicity or culture on experiences of care
‘Controversial’ comments about ethnic differences (NB these do not include comments clearly intended to be humorous)	**Description:** Comments or descriptions that might suggest racial discrimination.
	**Evidence:** E.g. Comments about one ethnic group receiving preferential treatment because of their ethnicity.
References to external factors that may influence service quality	**Description:** References and descriptions to external factors that may impact on service quality.
	**Evidence:** E.g. Comments about financial restraints, cutbacks or limited funding.
Emphasis on providing the best care irrespective of ethnicity	**Description:** References to the importance of good quality care.
	**Evidence:** E.g. Comments about care workers ‘doing the best they can’.
Sharing personal/difficult or emotional stories	**Description:** Descriptions of difficult experiences and emotions.
	**Evidence:** E.g. Descriptions of negative emotions resulting from their caring situation; negative descriptions of the stroke survivor.

## Results

There were seven focus groups lasting on average 80 minutes (from 50 to 90 minutes). Groups ranged in size from three to eight participants and were conducted in community venues. Locations were selected to be convenient, comfortable and familiar to participants [4].

Table 2 shows the demographic characteristics of the focus group participants. Five of these were categorised as more ethnically homogenous groups. Two included only White British participants, two only South Asian participants and one included only participants who were Black African or Black Caribbean. The remaining two focus groups, designated more heterogeneous, included a mixture of participants from White British, Asian and Black African or Black Caribbean groups. There were a total of 41 participants ranging in age from 45 to 74 years. Most were over 50 years (n = 33) and were spouses (n = 28). They had been carers for between 14 months and 20 years. Approximately two-thirds (n = 29) were female. None of Black and minority ethnic (BME) participants were born in the United Kingdom (UK) but all White British participants were.

**Table 2:** **Participant demographics: numbers of participants in each group**

Focus groups	Age category		Gender		Relationship	
More homogeneous groups						
	< 50 yrs	50 + yrs	Female	Male	Spouse	Other
**Group A**	2	5	5	2	6	1
Asian Indian = 5						
Asian Pakistani = 2						
**Group B**	1	6	4	3	2	5
Asian Indian = 7						
**Group C**	0	5	2	3	4	1
White British = 5						
**Group D**	0	3	3	0	3	0
White British = 3						
**Group E**	3	4	5	2	2	5
Black Caribbean = 3						
Black African = 4						
**More heterogeneous groups**						
**Group F**	0	5	3	2	4	1
Asian Indian = 1						
Asian Pakistani = 2						
Black Caribbean = 1						
White British = 1						
**Group G**	2	5	7	0	7	0
Asian Indian = 4						
Black African = 1						
White British = 1						
Black Caribbean = 1						
**Total = 41**	8	33	29	12	28	13

The focus groups were lively and participants frequently remarked how much they had enjoyed themselves and had learnt from others. In all groups there was considerable consensus and relatively few challenges. The shared bond of being a carer was clear although its prominence varied between groups. Participants often offered each other support and validated their caring roles. Emotional discussions about the challenges of being a carer and difficulties negotiating services led to animated, supportive discussions where participants offered each other advice on local services and made suggestions of how to manage their sometimes demanding role.

### Comparison of more homogenous focus groups with more heterogeneous groups

The following section compares data from the five more ethnically homogenous focus groups with data from the two more heterogeneous groups. Where relevant, a distinction is made between the White British focus groups and those with only minority ethnic participants. Findings are described under three main sections with references to how they fit with the analytical framework (Table 1):

1. Responses to a direct question about ethnicity and satisfaction with services.
2. Apparent comfort in discussions about ethnic and cultural differences.
3. Difficult experiences and emotions as carers.

Participants have been given pseudonyms. Their ethnicity is identified as follows: Black African (BA); Black Caribbean (BC); Asian Indian (AI); Asian Pakistani (AP); White British (WB).

#### Responses to a direct question about ethnicity and satisfaction with services

Towards the end of the focus group participants were directly asked whether they thought user ethnicity might be related to satisfaction with social care services. Reactions varied but there were clear differences between the more and less ethnically homogenous groups. In summary, four of the five more homogenous focus groups (two Asian, one Black African/Black Caribbean and one White British) generally agreed that user ethnicity might influence satisfaction. In contrast, participants in the two more heterogeneous groups appeared not to think there was a relationship.

### The more homogenous focus groups

There was frequently overt consensus in saying that user ethnicity might influence satisfaction in the more homogenous BME focus groups. Participants often cited language differences, lack of information and service providers’ limited understanding of cultural and religious practices as possible explanations. Expressions of agreement were common with phrases such as ‘Yes, exactly’ (Table 1). For example in one Asian group:

**Amiya** (AI): ‘Yes it does, yes it does. I’m different because I’ve been brought up in this country. I’ve been here most of my life. But yes, it does.’

**Sathinder** (AI): ‘It makes a lot of difference… It’s a language barrier, it’s a, you know, they can’t explain themselves because they … like some of the people can’t speak English as such… But still to explain themselves, to express their feelings about things… they don’t know. They don’t know where to start with, they don’t know who to ask or what to do.’

The Asian Indian group gave specific examples of difficulties associated with lack of understanding of religious and cultural differences. Care workers from their own religion were preferred. Here too there was a lot of consensus.

**Khayrah** (AI): ‘Yeah, exactly. So they don’t know about, they don’t know about our needs, our religious needs and stuff like that. So obviously like, when she’s trying to do her ablution, like she constantly has to explain to them, like, what she needs to do and like they can’t understand, they think it’s a bit stupid or a bit petty, or something. … It’s like, you get like, certain social workers that do respect, um, your background and your beliefs and things like that. But then you get some that just, like, you know they just think ‘Oh well’, you know, they’re not bothered.’

**Tanweer** (AI): ‘You’re just making a fuss.’

**Khayrah** (AI): ‘Yeah, yeah, you’re just making a fuss over nothing, do you know what I mean?’

**Adeeba** (AI): ‘And food as well. You know. So nobody can prepare my food like, you know. So this is an issue…’

There was also consensus in the Black African/Black Caribbean focus group about other aspects of communication. Lea, a Black Caribbean carer commented that the way someone from the Caribbean spoke might be perceived negatively. Sarah agreed.

**Lea** (BC): ‘Actually, right (*laughs*). Actually it’s not just from the different culture but from Black people as a whole. To an English person, an English person might think sometimes when we talk to them, we are shouting. We are not shouting. It’s the way we were brought up, we are loud. We talk much louder. So with an English person who is not used to a Black person, or Black people, they might think ‘Oh, she is shouting at me’. We are not shouting.’

**Sarah** (BA): It might come across as aggressive.

There was general consensus here about the impact of ethnicity on perceptions of services although the idea service providers should take cultural differences into account was directly challenged although Sarah emphasised that this was her opinion.

**Sarah** (BC): ‘I’d like to differ. If you are living in this country here for 50 years or more, I don’t think that you should expect the same culture as when you left home all those years ago. I think you are blended into the English culture, so that is me personally, that’s the way I would think.’

However, the group eventually resolved the discussion acknowledging that more than one viewpoint was possible:

**Nancy** (BA): ‘I think I am speaking from the point of view when my mum here, so she is new in the country, and how the helper came and how she was managed, so she wasn’t satisfied. But erm, later on when my sister came and started helping, it was quite a different thing. So I am speaking from the point of view of my own experience and the little time she was in this country…’ (laughs)

**Sarah** (BC): ‘You’ve got to look at it both ways.’

In one White British group there was consensus that user ethnicity might be related to service satisfaction. However, unlike the BME groups, they did not discuss language differences instead they focussed on societal perceptions of cultural differences in how families support each. Since BME users were believed to want to look after each other, fewer services might be offered to them, potentially leading to service dissatisfaction. However, Mary acknowledged her lack of direct evidence.

**Mary** (WB): ‘You find most Asian people they have, they will have Grandma and Grandpa living with them.’

**Barbara** (WB): ‘Yeah.’

**Mary** (WB): ‘And coping with them. I mean when I was a kid, you always had Nan and Grandpa there, you know, and you looked after them. But you don’t now, everybody has moved away. So I think perhaps the Asian people might respond differently. But I don’t know. Supposition.’

In contrast, the other White British group took a different stance and discussed how White British service users’ experiences and perceptions of services might potentially be adversely affected by care worker ethnicity.

**Graham** (WB): ‘Initially possibly. Um because I mean the underlying criteria is that you want the best care for your wife, for your loved one, for your carée. Umm… and somebody turns up at the door and whether they are Black or White or whether they speak English or not. Well that not’s true but how well they speak English, you’re going to make an instant appraisal and you’re going to watch and see. But I have found that everybody is a person to me.’

### The more heterogeneous groups

In contrast to the more homogenous groups, there was general consensus in the two more heterogeneous groups that ethnicity would not influence service satisfaction. Participants stressed issues important to all service users, irrespective of ethnicity. The first more heterogeneous group highlighted the importance of being treated as an individual irrespective of ethnicity whilst the second group emphasised external factors and general service issues affecting all users such as financial constraints (Table 1). Here too the discussion focussed on issues common to all users and away from ethnicity.

In the first heterogeneous group Peter suggested that, although professionals can be trained in cultural differences, wider issues and the pressure social care staff are under are more important.

**Peter** (BC): ‘I think it’s the problem of trying to understand the needs of people from ethnic minority. … But I still think that at the end of the day, despite all that little bit of understanding that they’re having, it comes back down to finance and lack of communication. As Helen said, this is another factor of people who are working in the service they are under such pressure…’

Participants in the other heterogeneous focus group suggested receiving caring, respectful, good quality services was more important than user ethnicity (Table 1). Again there was consensus evidenced by people talking to each other and recorded by the research team.

**Usha** (AI): ‘… caring is caring, and if it's done properly then, you know.’

**Ana** (AI): ‘That's the best doctors, the best nurses.’

**Freda** (WB): ‘All people respond to kindness. (*General agreement*) Especially if you’re in a weak position, you’re in hospital or just come out of hospital.’

However, one dissenter challenged the group and tried unsuccessfully to encourage further debate on the topic but the group appeared unwilling to discuss it further and the conversation ended abruptly.

**Rosa** (BC): ‘I think there’s an element, in ethnic background…’

**Ana** (AI): ‘You think so?’

**Rosa** (BC): ‘Yes.’

**Ana** (AI): ‘OK.’

#### Apparent comfort in discussions about ethnicity and culture

In this section possible indicators of feeling comfortable or at ease discussing ethnic and cultural differences are described. It is assumed that participants who are more comfortable discussing the topic will be more likely to mention ethnicity spontaneously and to discuss issues about ethnicity in depth. Potentially controversial comments are also described. Consensus and challenges are also highlighted here but there were no clear patterns across the groups in either challenges or consensus in terms of ethnic homogeneity or heterogeneity of the groups.

### Unsolicited references to ethnic and cultural differences

Participants in all groups made some spontaneous comments about ethnic and cultural differences (Table 1) but groups varied in terms of timing (when they were first made), number and content of comments.

### The more homogenous focus groups

In more homogenous BME groups, references to ethnicity and culture were generally made sooner than in the White British groups. When ethnicity was spontaneously mentioned, it was often in relation to Direct Payments. For example, in the Black African/Black Caribbean group, Abeje mentioned her ethnicity when introducing herself:

**Abeje** (BA): ‘… after the stroke he lost some of his English, you know, he speaks mainly Igbo, and Italian, because he lived in Italy … So, when Direct Payment was introduced, things became a lot easier because I had to employ carers from my Nigerian background, who were especially Igbo.... So, with Direct Payment I can actually tell the carer to stay an hour and a half, or two and a half hours. You know, give him simple massage. Like in our own culture there is a particular cream we believe so much that when you use it to massage people that had stroke, erm, it’s called er… Shea butter that’s it. So, some people that are not from an African background might not understand it. The cream, maybe they won’t like to touch it, because it’s not very good looking…’

Abasie agreed with her:

**Abasie** (BA): ‘… You can actually hire somebody who understands your background, culture, religion if you like. That really helped a lot. Because most times when carers come to see Mum, Mum doesn’t understand them. And they don’t know her culture, or her language and all that. And, but right now, she’s a lot happier.’

Early on in one Asian group, Omar spontaneously mentioned cultural differences.

**Omar** (AI): ‘… Because the majority of us here, we’re of an Asian group, we’re Muslims, so we do look after our elderly anyway at the end of the day. It’s only in extreme circumstances that, er, that they’ll be put in to care homes if you can’t cope.’

Although the other Asian group (a mixture of Asian Indian and Asian Pakistani participants) did not spontaneously mention ethnic differences until after a direct question from the moderator, once they did talk about it, there was a lot of discussion about why Asian users might be less satisfied with services including language differences. However, they agreed that having ethnicity in common with a social worker did not always guarantee satisfactory support.

**Sathinder** (AI): ‘I had to report her, I’m sorry, she was a Punjabi lady and she was something in the council and I’ll never forget her. I was so furious! And I said ‘Don’t talk to me like that because I work in a hospital and I know more than you know so don’t you ever treat me like this.”

**Samiya** (AI): ‘She said to me to put my Dad in a nursing home because I just broke down and said ‘I can’t cope. My Mum’s gone on holiday and I manage my dad 24/7. Can I have some help?’ She said, ‘Residential nursing home. Stick him in there.’ And I said, ‘I can’t do that’ and I was crying and crying and she just said, ‘Well, there’s nothing that I can do for you.”

In contrast, in both White British groups, ethnicity was not mentioned until quite late into the discussion and when it was, participants did not dwell on it. In one group ethnicity was not brought up until after the moderator’s direct question about ethnicity. In the other group, although mentioned earlier, it was hearsay, not personal experience:

**Graham** (WB): ‘… I hear stories and you speak to some carers and they say, ‘Well, other people who use social services don’t know who they are going to get. Are they going to turn up? I mean they all speak English but they’re not English people very often because they don’t pay very much.’ … But it, but they’re good. One Romanian and one Hungarian and a back-up Estonian. Sad isn’t it really? But they’re good.’

No one followed up on these comments but later Martin highlighted the fact that their care workers were African but repeatedly emphasised how good they were:

**Martin** (WB): ‘… and our experience of them over the past three or four months on the whole, has been positive. … Communication is good and in terms of, you know, I think they do work very hard to provide a reliable service. So we have Africans 115 hours a week in our home (*laughs*).’ … ‘But on the whole we have three… there’s been three, quite good continuity of carers. So our night time care is pretty much always done by three African ladies. And they’ve become part of the family. I mean we love them and they love us.’

### The more heterogeneous focus groups

In one of the more ethnically heterogeneous groups, ethnic differences were discussed early on but amongst the Asian participants only. Here Chetna emphasised the value she placed on the informal support she received from her Indian neighbour. Another South Asian participant, Abdul agreed:

**Chetna** (AP): ‘Just like family you know? So we living last 31 years together. So anytime we need each other’s help we can …’

**Abdul** (AI): ‘You know what I’m saying if people come from your own religion (I’m from Pakistan) if they come from your own religion they understand and we can trust the as well. But not everybody. Uh, but if it comes from the different culture it’s different …’

However, the second more heterogeneous group stood out from all other groups for having so few references to ethnic or cultural differences. When these were mentioned, they were as asides (e.g. a carer described how she translated her husband’s request in Guajarati to an occupational therapist). Discussions on the topic were generally brief.

### Potentially ‘controversial’ comments

There were a few, rare comments about ethnic or cultural differences which we have described as ‘controversial’ (Table 1). These refer to comments that might be perceived as a reflection of racial discrimination by services. Strikingly such comments were only made in the more homogenous groups with none in the more heterogeneous groups. Sometimes there was general agreement as with the small White British group but in other groups these were openly challenged, for example the Black African/Black Caribbean group.

The following is an example from a White British group. The consensus was very clear amongst the three women.

**Mary** (WB): ‘And they sent a doctor down to assess Mike, refill this form in, he was an Asian man, and he come to the end of it, and he looked at Mike and he said ‘Well there’s two things wrong.’ He said ‘One, the pot’s running out of money.’ He said ‘Secondly your skin’s the wrong colour.’ And I thought ‘Well that’s great coming from a coloured man!” [*laughter*].

**Barbara** (WB): Yeah.

**Mary** (WB): So in other words he was telling us that if we were dark, we’d have got the money, but because we were White, we weren’t likely to get it.

**Margare**t (WB): Yes.

**Barbara** (WB): ‘And I was told the very same thing about the hoist. And my doctor is a foreign doctor. I was told the very same thing. He said ‘I give you a letter saying you’re entitled to it and your physio turned you down. I can’t see why, because I wrote the letter. I’ve been in your house, I’ve seen the state your husband is in, and you’re turned down.’ He said ‘I can’t see why.’ He said exactly what you said: ‘The wrong colour.”

**Mary** (WB): ‘And I mean, fancy being told that by a doctor?’

In the other White British group, Graham admitted initial doubts about their care workers because of their ethnicity.

**Graham** (WB): ‘But I think to be honest with myself, yes, I do probably, to myself think ‘Are they going to be good enough?’ You can’t help but have a generalisation in your head about how people are from where they come. But we’ve had everybody. We’ve had enablement, we’ve had people from North Africa, people from Sudan, West Africa and India and they’ve been fine. As long as they are well trained and that over a number of months and years it becomes ingrained in you so you think ‘Well, if they have been trained doing it. Let’s keep an eye on them to start with and then let’s have trust.”

This was not challenged but John responded by suggesting the group should also consider other ethnic groups’ perspectives.

**John** (WB): I mean, if it as an Indian family would they be accepting… a White British person coming in to care for them? And I’m sure they would be exactly the same as us and I am sure if a British person was doing their job to the best of their ability, then I think that would be ‘A OK’. Well it certainly would be with me.’

One participant openly agreed with the following potentially controversial comment about immigration made in the Black African/Black Caribbean group was but not responded to by the rest of the group.

**Lea** (BC): ‘Personally, I think Britain - as we call it the Mother Country - Britain has tried to help everyone and anyone. And at the end of it now, I think they’ve taken on too much and they forget their own. And it’s time they knuckle down and think of their own backyard, before they going all over the place. That’s what made Britain go backward, and not enough of this and not enough of that. Because they’ve taken on too much.’

**Sarah** (BC): ‘How much can you take on? There’s a limit. There’s a limit to how much you can.’

In contrast, Samiya’s comment about the ethnicity of attendees at a council meeting in an Asian focus group was openly challenged by others and she later apologised.

**Samiya** (AI): ‘Because they were White people.’

**Hardit** (AI): ‘You can’t say that.’

**Samiya** (AI): ‘Sorry, sorry. Most people who are well-off they pay for their own personal care. Most Asians have difficulty so they have to pay something towards personal care but most of it comes from the council.’

Sabih from the other Asian focus group also appeared to suggest that he wondered whether, compared to other ethnic groups, White English users may receive different services. Tanweer acknowledged specific cultural issues whilst highlighting common concerns (Table 1).

**Sabih** (AI): **‘**… But I don’t know how well, sort of, the White English are treated, not being racist or anything. You know, they are not here to say the type of things they get which we don’t get or which we are not told you see. So that’s a bit of a problem as well there you see. ‘

… Because I mean I… I’m led to believe we all should have the same care of service.’

**Tanweer** (AI): ‘I think some issues probably go across the board with White communities and ethnic communities. I mean we have the culture, religion and language problems but there are some issues which go across the board.’

#### Describing difficult experiences and emotions

All groups shared difficult experiences with other participants but in contrast to discussions about ethnic differences, there were no clear patterns in terms of the ethnic group composition and their apparent comfort in sharing these experiences. Discussions about the challenges of being a carer and sharing emotional difficulties (Table 1) were common, often starting early in their discussions when introducing themselves.

### The more homogenous groups

The small White British group stood out as including a great many examples of references to difficult experiences. They appeared to develop a strong bond and shared many practical tips for looking after their husbands. Humour ran throughout their discussions.

**Mary** (WB): ‘But I have to go in with him in the bathroom, and tend to all his needs, and that’s not very nice for him. Luckily we both have a very warped sense of humour.’ [*laughter*]

**Barbara** (WB): ‘It helps.’

**Margaret** (WB): ‘It does. It definitely does.’

**Mary** (WB): ‘And we’ve got it down now to a fine art. We’ve got, um, a stair lift to bring him up the stairs so I take him in the bathroom. We’ve got one of these, I think they call them a tilt chair or something, sits up against the sink so he can wash himself. … But I find, keep bending up and down, that makes me feel sick… What our neighbours must think we get up to I don’t know. The other morning I was saying to him, ‘Oh I do feel sick.’ I said ‘Do you think I could be pregnant?’ And he’s saying to me ‘Well do you think it’s mine?’ [*laughter*] And we get fits of the giggles. And this is about quarter to seven in the morning.’

The other more ethnically homogenous groups described difficult experiences. Hardit was immediately supported by Sathinder:

**Hardit** (AI): ‘… And I was getting frustrated as well because I didn’t have the calmness and things like that, you know. But you have to change and I’ve changed so much. … She says things which she doesn’t… it just comes out. … So she fires at me and … I go upstairs and I cry you know. Sorry (*clears throat*). But uh, it’s not her fault what she’s going through but it hurts me you know.’

**Sathinder** (AI) ‘They don’t realise, this is the trouble.’

### The more heterogeneous groups

Occurrences of descriptions of difficult experiences were similar to those in the more homogenous groups and were discussed in a similar manner. The following quotes are examples from both the more heterogeneous groups. The first relates to cognitive changes in Helen’s husband and the second to the impact of being a carer.

**Helen** (WB): ‘You end up going the wrong, the wrong path and end up back at the beginning. And then he’ll say the opposite to ‘yes’ or ‘no’, whichever it was before, and you realise that you’ve spent 10/15 minutes trying to find out something that wasn’t…um (*laughs*). You say about people changing, I mean he was never a very, what shall we say? I can’t think of the right word. But he is now very grumpy and bad tempered. He used to get like that at times, but now he is like that all of the time. I think it’s mostly frustration…’

**Hyat** (AP): ‘My husband has stroke, 3 year. So, very, very hard time. So, if somebody is low and is sick like that, so. I have very short… I’m always crying and you know …’

## Discussion

Conversations in the seven focus groups were animated and participants appeared to enjoy themselves. Not surprisingly, given their caring role in common, groups often spontaneously discussed the impact of stroke and their experiences as older carers of stroke survivors. This similarity in experience may help explain the considerable degree of consensus and also the frequency with which participants offered each other support. However, at times they openly disagreed with each other.

We compared ethnically more homogenous and more heterogeneous focus groups to investigate whether there were differences between them when discussing ethnicity and culture and found that the ethnically more homogenous focus groups appeared more comfortable discussing ethnic and cultural differences than the more heterogeneous groups. There were differences both in how they responded to direct questioning on the topic and in the content of their discussions. For example, participants in the more homogenous groups made potentially controversial comments whilst participants in the more heterogeneous groups appeared to make more attempts to deflect the conversation away from ethnicity onto issues common to all ethnic groups. This suggests that the more ethnically homogenous groups were more comfortable discussing ethnic differences than the more heterogeneous groups. This provides evidence for the guidance offered previously [4, 5] which argues for participant compatibility. However it also advances our understanding and adds to the literature by showing potential ways to operationalise concepts such as being more or less comfortable with other focus group participants and providing specific suggestions of what evidence to look for in focus group data. It has been argued that discussions generated in focus groups are a mixture of collective narratives and personal beliefs influenced by local circumstances [19]. Here there were many references to external factors such as financial constraints negatively affecting service quality which may be a reflection of such narratives but may also be used by participants to move potentially uncomfortable discussions onto more general issues.

To further our understanding of the influence of focus group composition on interaction, we also looked at discussions concerning topics unrelated to ethnicity – here the impact of being a carer. No clear patterns were identified suggesting that ethnic group composition had less influence on such discussions. However, the fact that the small White British focus group, who were the most similar demographically overall (age, gender and relationship with stroke survivors), stood out as most comfortable in sharing their experiences and emotions as carers, suggests that demographic homogeneity in factors other than ethnicity may have had an impact on these discussions and supports Morgan (1998) who argues that ‘Whether a demographic characteristic will affect the compatibility of the participants depends on the topic of the research.’ ([5]:61).

When selecting focus group participants, it should not be assumed that data from more homogenous focus groups are ‘better’ or more informative than data from more heterogeneous groups. Data generated from these different sources should be seen as complementary. Both provide information of how issues around ethnicity and services are framed, perceived and discussed. For example, the emphasis in the more heterogeneous groups on the impact of being a carer of someone with stroke, individualised care and ensuring that all users receive the best possible care is an important point that needs highlighting. All too often the emphasis is on differing needs of users [20].

This paper relates to relative ethnic homogeneity and discussions around ethnicity but the ideas generated are likely to be transferable to homogeneity in other demographic characteristics and other topics. The fact that relative homogeneity in ethnic group composition appeared to have less impact on topics unrelated to ethnicity suggests that greater clarity in what is meant by homogeneity is required. Its importance will depend on the research question and the specific participants involved. Clearly even if homogeneity in one characteristic, for example gender, is possible multiple variations in other characteristics such as age, education and ethnicity mean homogeneous focus groups in multiple demographic terms may be unachievable. In addition, other, perhaps less easily definable, potentially unknown factors may affect group interaction. In our study, the shared experience in common of being a carer for someone with stroke created a clear bond between participants of both relatively homogenous and heterogeneous groups. This echoes Morgan 1998) [5] who reported that difficult experiences such as caring for someone with Alzheimer’s disease may override even close demographic similarities. We would therefore suggest that it may be wiser to refer only to homogeneity and heterogeneity in relative terms.

‘Sensitive moments’ in focus group discussions shown by participants’ hesitation, awkwardness, defensiveness or where they begin a cautious exploration of a topic but then retreat or are ‘pushed back into safer territory’ have been highlighted [21]. Such incidences are of particular interest here because paradoxically, they can occur when people are comfortable with each other. Such sensitive moments in our focus groups mostly coincided with what we described as the controversial comments which only occurred in more homogenous groups. For example, Nancy in the Black African/Black Caribbean group was challenged by another participant after suggesting services should take cultural factors into account. She then appeared to back track, distancing herself from her original comment by saying she was taking her mother’s perspective. This appeared to be an example of a participant retreating ‘back into safer territory.’

Our exploratory analysis highlights some of the potentially confounding issues when trying to understand focus group membership and interaction. Here we concentrated on homogeneity in ethnicity but participants’ ethnicity itself may have influenced both what and how participants talked about ethnicity. For example, probably because they were from an ethnic majority, the White British participants talked less about ethnic differences and were at times more likely to offer collective narratives [8] rather than personal experiences.

### Study limitations

This was an exploratory study with several limitations, some of which were largely beyond the researchers’ control and occur generally in focus groups. For example, some focus group participants appeared to bond more than others. Participants here were all older carers of stroke survivors but in order to be more confident in our comparisons, greater similarity in group size and gender balance would have been preferable. However, recruiting carers is acknowledged as challenging particularly if for example specific demographic characteristics are wanted [22]. Carers frequently find it problematic to leave the person they care for, but recruitment here was especially difficult as they had to be from specific ethnic groups, aged over 45 years and looking after stroke survivors. Furthermore recruiting participants from BME groups can be difficult because of lack of confidence in the English language, not understanding what research is and in being able to have anything useful to say [23].

The ethnic groups represented here were diverse and included people from different countries, generations and religions. Ideally we might have explored smaller, less diverse groups and would have run separate groups for the included ethnic groups. However, even broad terms such as ‘Asian’ and ‘Black’ were recognised by our groups as they spontaneously referred to them.

We were unable to investigate the impact of participant gender on the discussions. In multi-ethnic, mixed gender groups, women are more concerned with building rapport than men [24]. Perhaps supporting this, participants in the smallest group here which included only three White British women were the most overtly supportive and had a striking rapport with each other. However, this could be related to the small size of the group, and similarity in other features rather than that they were all women.

It is worth briefly considering the impact of social desirability [25] on participants’ responses and the relationship between social desirability and group homogeneity and heterogeneity. It may be that because of their apparent similarity, it was more socially desirable for participants in the more ethnically homogenous groups to make the controversial comments identified here. However, it is impossible to make definitive judgments about the impact of social desirability on this and since the responses to such comments from other focus group participants varied considerably with each groups, further exploration of this would be useful.

The moderators here were White British females giving continuity throughout the focus groups and facilitating data analysis [3]. Deliberate matching moderator ethnicity to participant ethnicity was considered but rejected for several reasons. For example, social class, gender and generational difference complicate ethnic identity, making selecting appropriate moderators almost impossible. Furthermore, advantages of dissimilar moderators include being able to naïve questions that someone from the participants’ group could not [5] whilst ‘sameness’ between researcher and researched can impede critical reflexive research [26].

## Conclusions

The multi-faceted nature of identity makes decisions about focus group participant selection extremely complex. Relative homogeneity in participants’ ethnic group membership influenced how participants talked about ethnic differences and how comfortable they appeared to be in discussions about this but it cannot be assumed that data derived from the more homogenous groups was more valuable than the data from the more heterogeneous groups. Rather the approaches provide different perspectives. Furthermore, depending on the topics under discussion, having characteristics in common, such as being a carer can override other differences. Our focus was on the impact of ethnic group composition on findings is very relevant to research in multi-cultural societies but has wider implications for designing and running focus groups in research not necessarily concerning ethnicity.

### RATS guidelines

The study conformed to the RATS guidelines [27].

## Abbreviations

BME: Black and minority ethnic
BA: Black African
BC: Black Caribbean
AI: Asian Indian
AP: Asian Pakistani
WB: White British
UK: United Kingdom.
